# Association between neutrophil–lymphocyte ratio change during living donor liver transplantation and graft survival

**DOI:** 10.1038/s41598-021-83814-9

**Published:** 2021-02-18

**Authors:** Jungchan Park, Seung-Hwa Lee, Mi Sook Gwak, Justin Sangwook Ko, Sangbin Han, Gyu-Seong Choi, Jae Won Joh, Jongman Kim, Gaab Soo Kim

**Affiliations:** 1 Department of Anesthesiology and Pain Medicine, Samsung Medical Center, Sungkyunkwan University School of Medicine, 81 Irwon-ro, Gangnam-gu, Seoul, 06351 Korea; 2 Division of Cardiology, Department of Medicine, Heart Vascular Stroke Institute, Samsung Medical Center, Sungkyunkwan University School of Medicine, Seoul, Korea; 3 Department of Surgery, Samsung Medical Center, Sungkyunkwan University School of Medicine, Seoul, Korea

**Keywords:** Biomarkers, Gastroenterology, Medical research, Risk factors

## Abstract

Preoperative neutrophil–lymphocyte ratio (NLR), has shown a predictive value in living donor liver transplantation (LDLT). However, the change in the NLR during LDLT has not been fully investigated. We aimed to compare graft survival between the NLR increase and decrease during LDLT. From June 1997 to April 2019, we identified 1292 adult LDLT recipients with intraoperative NLR change. The recipients were divided according to NLR change: 103 (8.0%) in the decrease group and 1189 (92.0%) in the increase group. The primary outcome was graft failure in the first year. In addition, variables associated with NLR change during LDLT were evaluated. During 1-year follow-up, graft failure was significantly higher in the decrease group (22.3% vs. 9.1%; hazard ratio 1.87; 95% confidence interval 1.10–3.18; *p* = 0.02), but postoperative complications did not differ between two groups. This finding was consistent for the overall follow-up. Variables associated with NLR decrease included preoperative NLR > 4, model for end-stage liver disease score, intraoperative inotropic infusion and red blood cell transfusion, and operative duration. The least absolute shrinkage and selection operator model yielded similar results. NLR decrease during LDLT appeared to be independently associated with graft survival. Further studies are needed to confirm our findings.

## Introduction

Neutrophil–lymphocyte ratio (NLR) is a simple, inexpensive, readily available, and reproducible index that reflects the systemic inflammatory respone^1^. The NLR has been shown to have a predictive value in various clinical situations including cardiovascular disease, cancer, and liver cirrhosis^2–4^. These findings were also consistently reported in surgical patients, and a perioperative high NLR was associated with adverse events after cardiac surgery and recurrence after cancer resection^5–10^.

Liver transplantation is an established treatment modality for end-stage liver disease and unresectable hepatocellular carcinoma (HCC) with or without cirrhotic change. Living donor liver transplantation (LDLT) provides a survival benefit as well as reduced waiting time for the candidates^11,12^. In liver transplant candidates, high NLR is a predictor of mortality^13^, and preoperative elevation of NLR also showed significant correlations with higher mortality and recurrence rate after liver transplantation for HCC^14,15^. Based on these previous findings, preoperative NLR was suggested to be helpful in selecting adequate HCC recipients for LDLT^14^. However, the current clinical value of NLR in LDLT seems to be limited to using preoperative value to predict outcome.

Postoperative NLR independently predicts mortality and adverse outcomes such as acute kidney injury and cardiovascular events in various surgeries and is associated with recurrence rate after cancer resection^8–10,16,17^. Furthermore, previous studies showed an efficacy of controlling intraoperative inflammation as indicated by the NLR to improve postoperative outcomes^18^. Therefore, we hypothesized that the change in NLR during LDLT could reflect operative burden and be associated with graft survival. In this study, we enrolled LDLT recipients with available NLR change data during the surgery and compared the incidence of graft failure. We also calculated the attributable fraction (AF) of each variable on graft failure and evaluated the variables that are associated with NLR change during LDLT using logistic regression and the least absolute shrinkage and selection operator (LASSO) models. Our finding may provide valuable information on the association between intraoperative NLR change and clinical outcome in LDLT and a direction for perioperative management and future studies.

## Results

### Baseline characteristics

**Figure 1:**
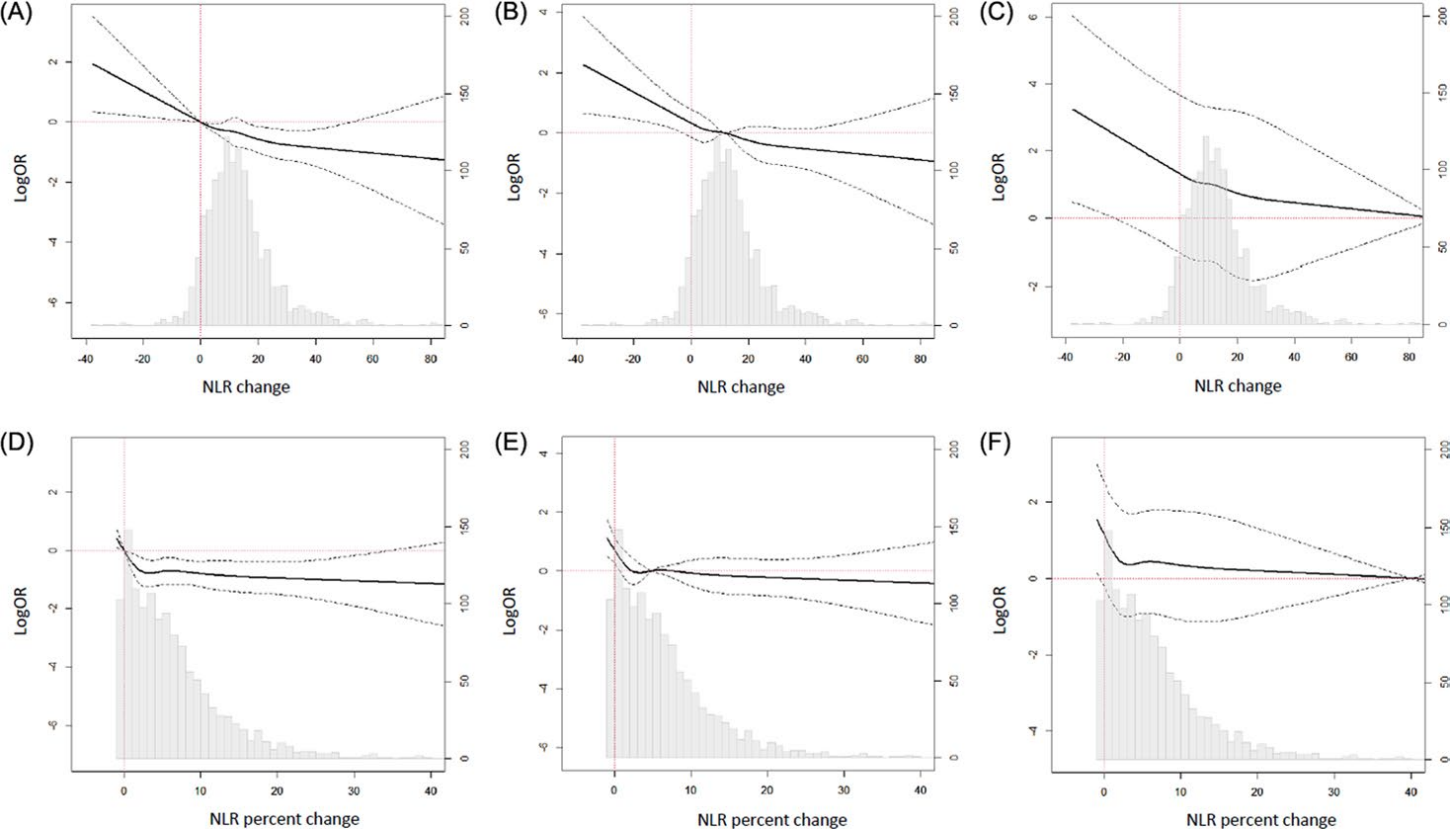
Smooth plots of the change of odds ratio for 1-year graft failure when the reference value was (**A**) absolute NLR change of 0, (**B**) median value of absolute NLR change, (**C**) 80% of absolute NLR change, (**D**) NLR percentage change of 0, (**E**) median value of NLR percentage change, and (**F**) 40% of NLR percentage change. (NLR percent change defined as [postoperative NLR − preoperative NLR]/preoperative NLR).

From June 1997 to April 2019, a total of 1349 cases of adult-to-adult LDLT were performed in our institution. From the entire cohort, 1306 cases of LDLT in which pre- and post-surgical NLR data were available were included, and 14 re-transplantation cases were excluded. Finally, 1292 LDLT recipients were enrolled for the study. Smooth plots for the change of odds ratio (OR) was construced and showed a significant association when the reference value was an absolute NLR change of 0 (Fig. 1), so we divided the recipients into two groups according to an absolute decrease or increase of NLR change during LDLT: 103 (8.0%) recipients in the decrease group and 1189 (92.0%) in the increase group. The baseline characteristics are summarized in Table 1. The decrease group had less males and was generally younger than the increase group. Those in the decrease group showed higher incidences of alcoholic use, hepatorenal syndrome, and encephalopathy. Those in this group also had longer preoperative intensive care unit (ICU) stays with higher model for end-stage liver disease (MELD) and Child–Pugh scores. Preoperative NLR value as well as the incidence of NLR > 4 was higher in the decrease group, but an absolute value of postoperative NLR was lower in the decrease group. For operative variables, the use of inotropic infusion was less frequent in the decrease group, and packed red blood cell transfusion was more frequent in the decrease group. The operative duration was longer for the decrease group.

**Table 1:** Baseline characteristics of the entire population according to the change of NLR.

	Decrease (n = 103)	Increase (n = 1189)	*p* value
**NLR level**			
Preoperative	13.96 (± 10.98)	3.46 (± 3.58)	< 0.001
Preoperative > 4	88 (85.4)	296 (24.9)	< 0.001
Postoperative	7.84 (± 6.44)	18.34 (± 12.85)	< 0.001
**Recipient variables**			
Age	48.3 (± 9.6)	52.5 (± 8.7)	< 0.001
Male	67 (65.0)	940 (97.1)	0.002
Body mass index	24.47 (± 3.88)	24.40 (± 3.41)	0.85
Smoking	20 (19.4)	190 (16.0)	0.44
Alcoholics	28 (27.2)	181 (15.2)	0.003
Hepatorenal syndrome	18 (17.5)	45 (3.8)	< 0.001
Encephalopathy	51 (49.5)	222 (18.7)	< 0.001
Varix	23 (22.3)	215 (18.1)	0.35
Ascites	68 (66.0)	679 (57.1)	0.1
Bacterial peritonitis	16 (15.5)	114 (9.6)	0.08
Hypertension	9 (8.7)	143 (12.0)	0.4
Diabetes	18 (17.5)	232 (19.5)	0.71
Tuberculosis	4 (3.9)	53 (4.5)	0.98
MELD score	29.2 (± 11.6)	16.9 (± 9.6)	< 0.001
Albumin	3.02 (± 0.52)	3.18 (± 0.65)	0.01
CTP score	11.0 (± 2.2)	8.5 (± 2.7)	< 0.001
Preoperative intensive care	35 (34.0)	69 (5.8)	< 0.001
**Pathology**			
Alcohol related	17 (16.5)	113 (9.5)	0.04
Viral	60 (58.3)	904 (76.0)	< 0.001
Cirrhosis	63 (61.2)	1092 (91.8)	< 0.001
Hepatocellular carcinoma	25 (24.3)	692 (58.2)	< 0.001
**Donor variables**			
Age	35.1 (± 11.1)	32.7 (± 11.4)	0.05
Male	57 (55.3)	776 (65.3)	0.06
Body mass index	23.51 (± 3.12)	23.25 (± 3.09)	0.42
Macrosteatosis	7.74 (± 6.91)	6.91 (± 6.22)	0.3
GRWR	1.07 (± 0.24)	1.08 (± 0.24)	0.9
**Operative variables**			
Right graft	101 (98.1)	1166 (98.1)	> 0.99
Inotropic infusion	40 (38.8)	777 (65.3)	< 0.001
Epinephrine bolus	11 (10.7)	167 (14.0)	0.42
Red blood cell transfusion	96 (93.2)	710 (59.7)	< 0.001
Operative duration, minutes	601.6 (± 155.5)	551.4 (± 117.1)	< 0.001

Values are n (%) or mean (± SD).

*NLR* neutrophil-to-lymphocyte ratio, *MELD* model for end-stage liver disease, *CTP* Child–Pugh score, *GRWR* graft to recipient-body weight ratio.

### Clinical outcomes

**Figure 2:**
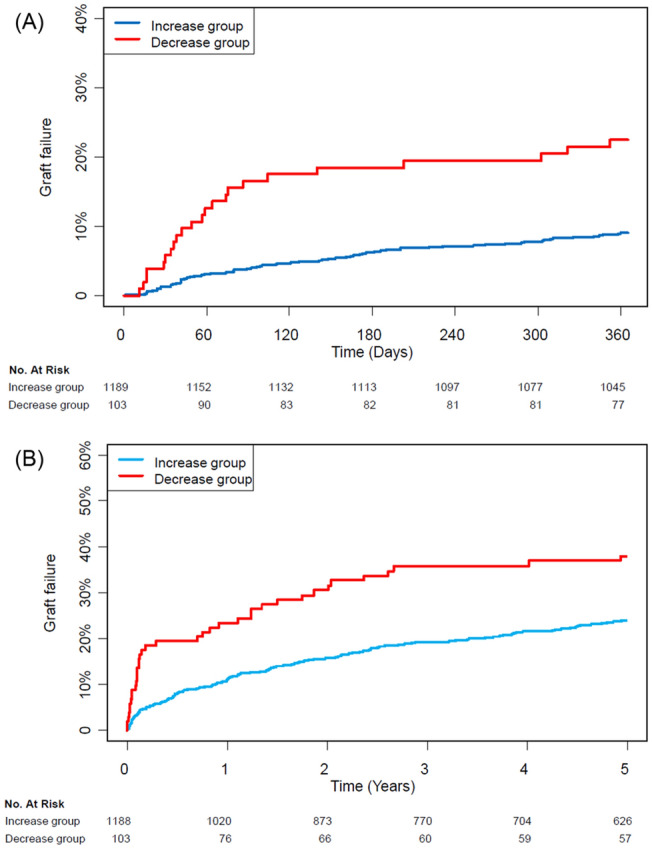
Kaplan Meier curves for graft failure during (**A**) 1-year follow-up and (**B**) overall follow-up.

**Figure 3:**
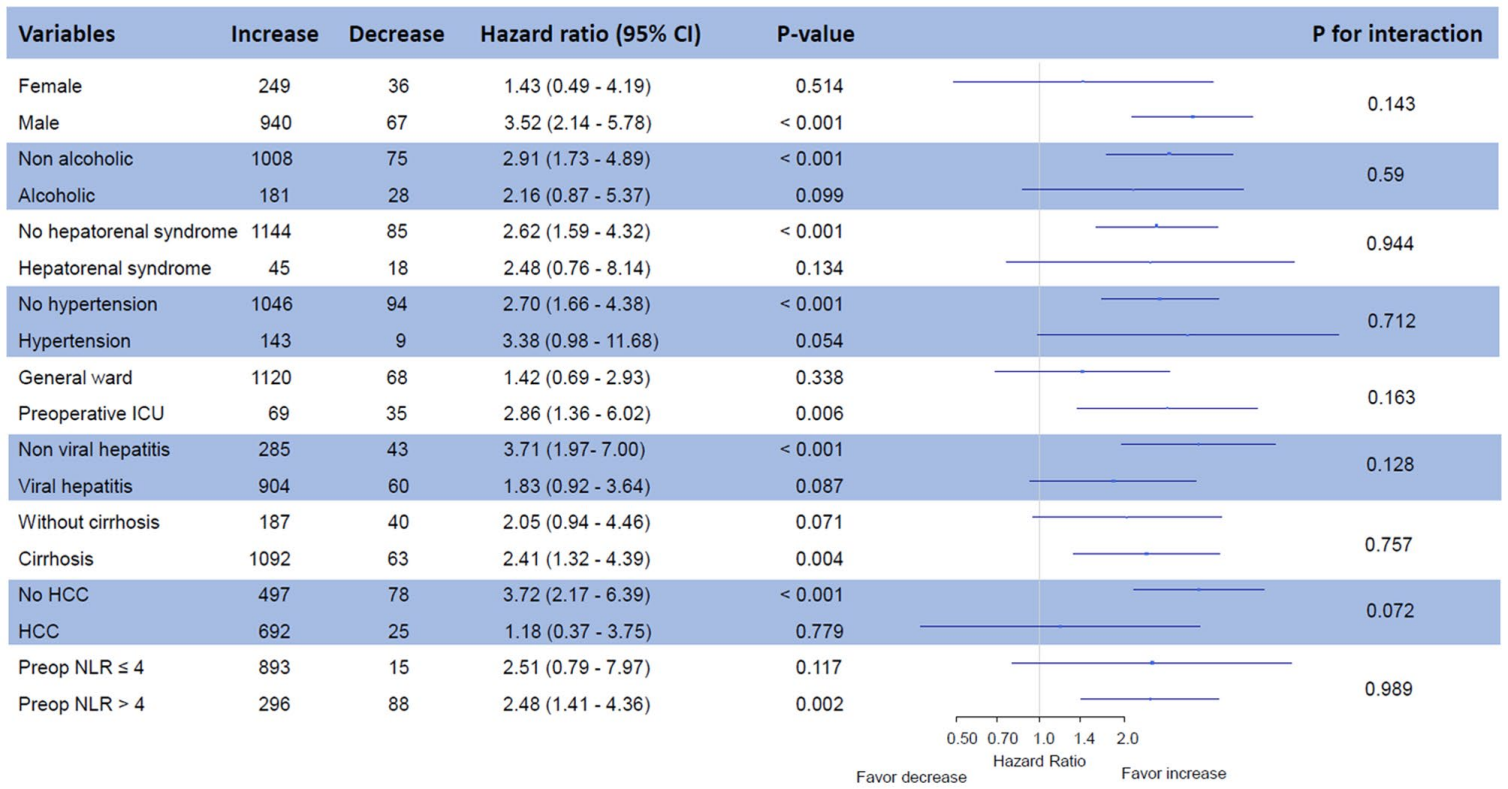
Forest plot for the subgroup analysis.

Clinical outcomes according to the change in NLR during LDLT are summarized in Table 2. For 1-year follow-up, the median durations were 365 [365–365] days in both groups. After adjustments, the incidence and risk of graft failure within 1 year were significantly higher in the decrease group [22.2% vs. 9.1%; hazard ratio (HR) 1.87; 95% confidence interval (CI) 1.11–3.21; *p* = 0.02] (Table 2) (Fig. 2A). The causes of graft failure in the both groups are summarized in Table S1, supporting information. The incidences of postoperative complication and graft rejection did not differ after adjustments during 1-year follow-up. A subgroup analysis revealed that there was no significant interaction (Fig. 3). The AF of significant variables on 1-year graft failure were 8.5% for NLR decrease and 14.5% for preoperative ICU stay (Table 3). In the sensitivity analysis, effects of an unmeasured confounder on the observed association was computed assuming that the prevalence of this confounder was 40%. The association was significant under all circumstances (Table S2, supporting information). Additionally, the observed association was significant before and after November 2010 (Table S3, supporting information).

**Table 2:** Clinical outcomes of the entire population.

	Decrease (n = 103)	Increase (n = 1189)	Unadjusted HR (95% CI)	*p* value	Adjusted HR (95% CI)	*p* value
**1-year follow-up**						
Graft failure	23 (22.3)	108 (9.1)	2.75 (1.76–4.32)	< 0.001	1.87 (1.10–3.18)	0.02
Death	23 (22.3)	108 (9.1)	2.75 (1.76–4.32)	< 0.001	1.87 (1.10–3.18)	0.02
Re-transplantation	3 (2.9)	8 (0.7)	4.83 (1.28–18.22)	0.02	4.08 (0.68–24.44)	0.12
**Postoperative complication**						
Grade IIIa–V	41 (39.8)	404 (34)	1.18 (0.86–1.63)	0.31	0.98 (0.69–1.40)	0.92
Grade IIIb–V	35 (34.0)	256 (21.5)	1.64 (1.16–2.34)	0.006	1.21 (0.81–1.80)	0.36
Grade IV–V	24 (23.3)	124 (10.4)	2.34 (1.51–3.62)	< 0.001	1.44 (0.87–2.37)	0.16
Graft rejection	1 (1.0)	25 (2.1)	1.03 (0.13–7.86)	0.98	1.42 (0.15–13.25)	0.76
**Overall follow-up**						
Graft failure	51 (49.5)	309 (26.0)	1.93 (1.43–2.60)	< 0.001	1.62 (1.15–2.29)	0.006
Death	48 (46.6)	287 (24.1)	1.98 (1.45–2.69)	< 0.001	1.72 (1.21–2.46)	0.003
Re-transplantation	10 (9.7)	44 (3.7)	2.41 (1.20–4.85)	0.01	1.97 (0.87–4.45)	0.1
**Postoperative complication**						
Grade IIIa–V	61 (59.2)	547 (46.0)	1.26 (0.96–1.64)	0.09	1.13 (0.84–1.52)	0.42
Grade IIIb–V	56 (54.4)	412 (34.7)	1.58 (1.19–2.09)	0.001	1.36 (0.99–1.86)	0.06
Grade IV–V	49 (47.6)	304 (25.6)	1.89 (1.39–2.55)	< 0.001	1.56 (1.20–2.21)	0.01
Graft rejection	28 (27.2)	183 (15.4)	2.08 (1.40–3.10)	< 0.001	1.93 (1.22–3.03)	0.005

Values are n (%) or mean (± SD).

*HR* hazard ratio, *CI* confidence interval, *IIIa* complications requiring interventions without general anesthesia, *IIIb* complications requiring interventions under general anesthesia, *IV* life-threatening complications, *V* death.

Variables for multivariable adjustment included age, sex, model for end-stage liver disease score, operative duration, alcoholics, hepatorenal syndrome, hypertension, preoperative intensive care unit treatment, viral disease, cirrhotic disease, hepatocellular carcinoma, preoperative neutrophil-to-lymphocyte ratio > 4, donor sex, and donor age.

**Table 3:** Attributable fraction of variables on 1-year graft failure after living donor liver transplantation.

	One-year graft failure/total recipients	One-year graft failure (95% CI)	Adjusted OR (95% CI)	Attributable fraction
NLR decrease	23/103	22.3 (14.4–32.7)	1.87 (1.10–3.18)	8.5
NLR increase	108/1189	9.1 (7.5–10.9)	Reference	
Preoperative intensive care	28/104	26.9 (18.1–38.2)	3.59 (1.93–6.66)	14.5
No preoperative intensive care	103/1188	8.7 (7.1–10.4)	Reference	
Cirrhosis	195/1155	16.9 (14.6–19.4)	1.23 (0.42–1.56)	− 4
No cirrhosis	26/137	19.0 (12.6–27.2)	Reference	
Hepatocellular carcinoma	72/717	10.0 (7.9–12.5)	1.53 (0.98–2.39)	17.1
No hepatocellular carcinoma	59/575	10.3 (7.9–13.1)	Reference	
Preoperative NLR > 4	50/384	13.0 (9.7–17.0)	0.97 (0.61–1.52)	− 1.2
Preoperative NLR ≤ 4	81/908	8.9 (7.1–11.0)	Reference	
Red blood cell transfusion	93/806	11.5 (9.3–14.0)	1.20 (0.77–1.86)	14.4
No red blood cell transfusion	38/486	7.8 (5.6–10.6)	Reference	

*NLR* neutrophil-to-lymphocyte ratio, *OR* odds ratio, *CI* confidence interval.

The attributable fraction is a measure that represents the proportional reduction in the incidence of graft failure within a population that would occur if the variable was absent, provided that a causal relation existed between that variable and the incidence of graft failure. We used incidence of variable and the association between the variable and the incidence of graft failure to calculate the attributable fraction.

For outcomes during the overall follow-up period, the median duration was 5.64 [2.01–10.40] years in the increase group and 7.04 [1.02–14.77] years in the decrease group (*p* = 0.54). As shown in the 1-year follow-up, the incidence of graft failure in the decrease group was higher during the overall follow-up period (49.5% vs. 26.0%; HR 1.62; 95% CI 1.15–2.28; *p* = 0.01) (Table 2) (Fig. 2B). Additionally, the composite of life-threatening complications and death during overall follow-up was significantly increased by NLR decrease during LDLT.

### Variables associated with NLR decrease during LDLT

**Figure 4:**
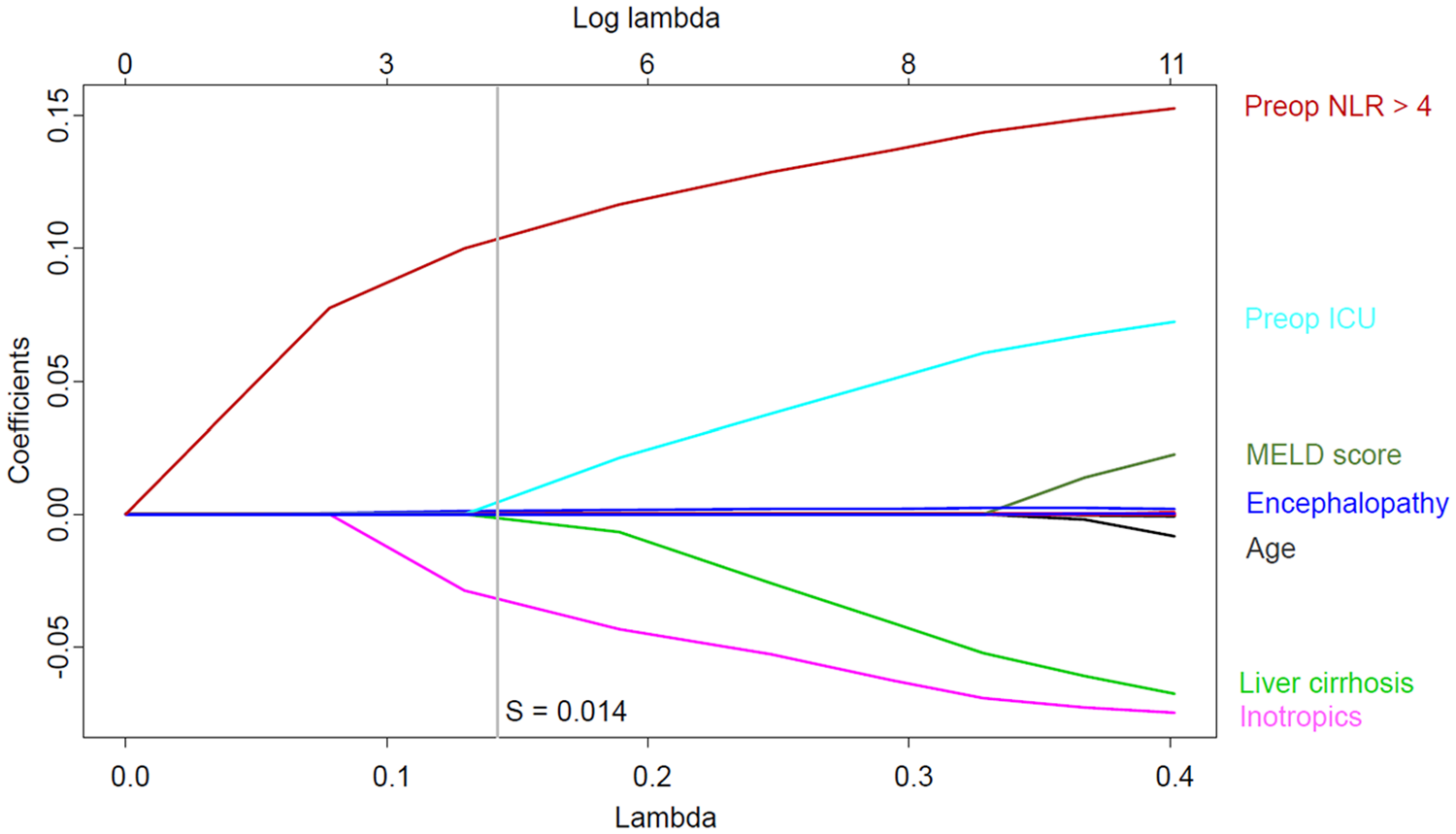
Variables associated with the decrease of neutrophil–lymphocyte ratio during living donor liver transplantation using the least absolute shrinkage and selection operator model.

In the logistic regression model, variables that were significantly associated with NLR decrease during LDLT included preoperative NLR > 4 (OR 9.17; 95% CI 4.92–18.09; *p* < 0.001), MELD score (OR 1.05; 95% CI 1.01–1.09; *p* = 0.02), no use of inotropic continuous infusion (OR 0.23; 95% CI 0.13–0.39; *p* < 0.001), intraoperative packed red blood cell transfusion (OR 2.71; 95% CI 1.67–7.20; *p* = 0.03), and operative duration (OR 1.00; 95% CI 1.00–1.01; *p* < 0.001) (Table 4). The LASSO model showed similar results to the logistic regression analysis and added that age, encephalopathy, cirrhotic disease, and preoperative ICU stay were also associated with NLR decrease (Fig. 4).

**Table 4:** Variable associated with NLR decrease during living donor liver transplantation.

	Adjusted OR (95% CI)	*p* value
Preoperative > 4	9.17 (4.92–18.09)	< 0.001
**Recipient variables**		
Age	0.99 (0.96–1.01)	0.26
Male	0.77 (0.41–1.47)	0.42
Body mass index	1.06 (0.92–1.10)	0.88
Smoking	0.99 (0.47–2.00)	0.97
Alcoholics	0.92 (0.46–1.81)	0.82
Hepatorenal syndrome	0.80 (0.32–1.94)	0.63
Encephalopathy	1.47 (0.76–2.81)	0.25
Varix	1.15 (0.60–2.15)	0.67
Ascites	0.79 (0.42–1.50)	0.46
Bacterial peritonitis	1.25 (0.58–2.64)	0.56
Hypertension	0.78 (0.30–1.83)	0.59
Diabetes	1.30 (0.66–2.50)	0.44
Tuberculosis	1.60 (0.44–4.64)	0.43
MELD score	1.05 (1.01–1.09)	0.02
Albumin	0.86 (0.49–1.50)	0.6
CTP score	1.00 (0.76–1.32)	0.99
Preoperative intensive care	1.32 (0.60–2.87)	0.48
**Pathology**		
Alcohol related	1.29 (0.54–3.04)	0.56
Viral	0.73 (0.39–1.39)	0.33
Cirrhosis	0.83 (0.40–1.76)	0.62
Hepatocellular carcinoma	0.72 (0.38–1.35)	0.31
**Donor variables**		
Age	1.01 (0.99–1.03)	0.44
Male	0.81 (0.47–1.40)	0.44
Body mass index	1.04 (0.95–1.12)	0.37
Macrosteatosis	1.01 (0.98–1.05)	0.54
GRWR	0.78 (0.19–3.15)	0.73
**Operative variables**		
Right graft	2.00 (0.37–17.56)	0.47
Inotropic infusion	0.23 (0.13–0.39)	< 0.001
Epinephrine bolus	1.99 (0.80–4.68)	0.12
Red blood cell transfusion	2.71 (1.16–7.20)	0.03
Operative duration, minutes	1.00 (1.00–1.01)	< 0.001

Values are n (%) or mean (± SD).

*NLR* neutrophil-to-lymphocyte ratio, *OR* odds ratio, *CI* confidence interval, *MELD* model for end-stage liver disease, *CTP* Child–Pugh score, *GRWR* graft to recipient-body weight ratio.

## Discussion

The main findings of the present study were: (1) the NLR value decreased during LDLT in 8.0% of the recipients, (2) the incidence of graft failure was significantly higher in the recipients with NLR decrease during LDLT, and (3) NLR decrease during LDLT was associated with preoperative variables such as NLR > 4 and MELD score and intraoperative variables such as use of inotropic infusion, packed red blood cell transfusion, and operative duration. These findings indicate that the change in NLR during LDLT may be associated with pre- and intraoperative conditions and may independently predict graft failure regardless of an absolute value.

Several biomarkers have been identified to detect and measure systemic inflammation, most of which consume additional time and cost^1^. NLR has the advantage of being inexpensive and readily available by daily measurements, and substantial evidence supports the association with clinical outcomes of acute and chronic conditions^1,19–21^. In previous studies on surgical patients, postoperative elevation of NLR has consistently shown associations with higher incidence of complication in cardiac and non-cardiac surgeries and recurrence after cancer surgeries^8–10,16^. Because there has been no previous report regarding postoperative NLR in LDLT, we initially evaluated the change of risk for graft failure according to postoperative NLR and different reference values of NLR change during LDLT. Based on our initial analysis, the recipients were divided into the increase and decrease groups by using dynamics of NLR during the surgical procedures instead of using an absolute value of postoperative NLR. Indeed, inflammatory markers have shown limited clinical utilities when using an absolute value in the postoperative period because an inflammatory response during the surgical procedure is inevitable. This response is characterized by a rise in circulating neutrophils accompanied by a fall in lymphocytes^22^.

In this study, we evaluated the change of NLR during surgical procedures instead of an absolute value and demonstrated that decreased NLR during LDLT was associated with increased incidence of graft failure despite the lower absolute value of postoperative NLR. Our explanation for this result is that, the increase of NLR could be regarded as a normal reaction to surgical injuries^23,24^, and the decrease of NLR during the surgical procedure, on the other hand, may be a surrogate marker that indicates generally poor condition of recipients who could not adequately respond to metabolic stress from LDLT procedures. Indeed, hematologic abnormalities are known to be associated with poor prognosis in cirrhotic patients^25^, and the cause of graft failure also supports our hypothesis. Compared with the increase group, the incidence of bleeding associated with graft failure was higher for the decrease group, and more graft failure in the decrease group was caused by pulmonary and cardiac complications, which are more closely related to general condition of patients, rather than graft rejection or surgical complication. However, determination of the degree of NLR increase that could be considered as normal requires further investigation. This is also true for the determination of whether an enormous increase in NLR is associated with adverse outcomes.

In addition to the aforementioned explanation on cirrhotic recipients, a separate explanation may be needed for the recipients with non-cirrhotic HCC. The prognostic impact of inflammatory response has discrete underlying mechanisms for cirrhotic disease and HCC. In cirrhotic disease, the alterations in the intestinal barrier along with increased luminal aerobic gram-negative bacilli, bacterial translocation, bacteremic events, and endotoxemia lead to increased inflammatory cytokine production which has prognostic significance^26^. In contrast, the effect of the inflammatory response on carcinogenesis plays a key role in the reoccurrence of HCC^15^. In previous studies, high NLR was associated with mortality of the overall liver transplant candidates^13^. However, in the recipients who actually underwent transplantation, preoperative NLR was mainly associated with outcomes of liver transplantation for HCC^14,15^. In the subgroup analysis, no significant interaction was observed for the association between NLR decrease and graft failure; however, considering a low incidence of NLR decrease, a larger study is needed to evaluate the generalizability of this finding.

Preoperative NLR elevation seems to play a complex role in our analysis. Preoperative NLR elevation was a strong predictor of NLR decrease during LDLT, but the calculated AF on graft failure was not significant in the multivariable model after retaining the intraoperative NLR decrease. Variables associated with NLR decrease during LDLT also included MELD score, supporting our explanation that the progression of disease and underlying state of the recipients may be involved in this association. Of note, intraoperative use of inotropic infusion and packed red blood cell transfusion also appeared to affect the incidence of NLR decrease. This finding suggests the possibility that adequate intraoperative management could prevent NLR decrease, but this requires further investigation. The control of perioperative inflammation resulted in improved outcomes of various cancer surgeries^18^, but whether prevention of intraoperative NLR decrease could improve outcome in LDLT or whether postoperative immunosuppression should be adjusted in the recipients with postoperative NLR decrease also requires investigation.

Our results should be appraised considering the following limitations. First, as a single-center retrospective study, our results may have been affected by confounding factors. Although relevant variables were adjusted for, the effect of unmeasured variables may have remained. A well-designed, prospective study with a larger number of patients may be needed to confirm our finding. Also, due to the long study period, advancements in surgical techniques and postoperative management could also have biased the results. Another limitation is that due to the low incidence of NLR decrease during LDLT, our results may have been biased despite the statistical adjustments and the larger number of the recipients than previous studies. Despite these limitations, this is the first study to demonstrate the negative impacts of NLR decrease during LDLT.

## Conclusion

The incidence of graft failure was significantly increased in the recipients with a NLR decrease during LDLT. Further studies are needed to confirm our findings.

## Methods

### Study population, data collection, and study endpoints

This retrospective observational cohort study was approved by the Institutional Review Board at our institution (Samsung Medical Center 2019-12-144) and was conducted according to the Declaration of Helsinki. The need for written informed consent was waived by the Institutional Review Board at our institution considering the minimal risk for the participants and retrospective nature of the study.

We reviewed the entire cohort of liver transplantation at our institution between June 1997 and April 2019 and identified adult-to-adult LDLT recipients. Cases were required to have available NLR data for pre- and postoperative period. This data were required to be obtained between before the anesthetic induction and immediately after arrival into the ICU. In recipients with multiple liver transplantations, only the first transplantation was enrolled for analysis. In the enrolled recipients, smooth plots of the change of OR for 1-year graft failure were constructed according different values of an absolute NLR change and NLR percent change, defined as (postoperative NLR − preoperative NLR)/preoperative NLR. According to the results of smooth plots, recipients were divided according to an absolute NLR change value of 0 into NLR increase or decrease groups. Clinical, laboratory, and outcome data were collected by a trained study coordinator who was not otherwise involved in this study, and NLR and its change were calculated by another investigator who was blinded to clinical outcomes. The data from all recipients were analyzed anonymously.

The primary endpoint of this study was graft failure during 1-year follow-up. Graft failure was defined as death or re-transplantation, and recipients who underwent re-transplantation before death were regarded as both death and re-transplantation in the table. One secondary endpoint was graft failure during the overall follow-up. Another secondary endpoint was the composite of complications according to Clavien–Dindo classification and graft rejection during overall follow-up. The classification was briefly defined as follows: IIIa, complications requiring interventions without general anesthesia; IIIb, complications requiring interventions under general anesthesia; IV, life-threatening complications; V, death^27^. Graft rejection was confirmed by biopsy when clinically suspected.

### Donor selection and surgical procedures

Donor selection criteria and surgical procedures of our institution have been previously described^28^. In brief, our criteria included adult younger than 65 years old, a body mass index lower than 35, biochemistries within normal range, and adequate size of graft and expected remnant liver of more than 30%. The presence of any conditions related to increased risk to the donor were excluded.

Most of the grafts were from the right side and consisted of 5 to 8 segments according to the Couinaud’s classification system. The surgical margin of the graft was determined after considering anatomical characteristics. For donors, full mobilization of the liver was achieved by dissecting the ligaments around the liver, and cholecystectomy was performed. After identifying bifurcations of the hepatic duct, portal vein, and hepatic artery, the intraparenchymal hepatic vein was identified with the aid of intraoperative ultrasound. The bile duct was transected after completing parenchymal dissection, and the graft liver was removed.

Harvested graft liver was implanted into the recipient using a piggyback technique. The right hepatic vein was initially anastomosed; if necessary, anastomosis of the inferior hepatic vein followed. After portal vein anastomosis, the hepatic vein and portal vein were unclamped to reperfuse the graft liver. After reperfusion, segment veins were anastomosed to the inferior vena cava using a cryopreserved allovascular graft, and the hepatic artery and biliary tract were then anastomosed. All recipients were routinely transferred to the ICU.

### Anesthetic care and perioperative management

Standardized anesthesia according to the institutional protocol was performed in all recipients. Under the standard monitoring of vital signs (peripheral capillary oxygen saturation, 5-lead electrocardiography, and non-invasive arterial blood pressure), the induction of anesthesia was achieved using thiopental sodium and maintained with isoflurane titrated to a bispectral index of 40 to 60. The radial artery, femoral artery and vein, and internal jugular vein were cannulated for direct hemodynamic monitoring and blood tests. The initial arterial blood gas analysis and complete blood cell test were performed before the surgical incision and repeated throughout the surgical procedure. Mechanical ventilation was set with a tidal volume of 8 to 10 mL/kg based on ideal body weight using a mixture of medical air and oxygen at a fresh gas flow rate of 2 L/min, and respiratory rate was continuously adjusted to maintain normocapnea. Remifentanil was used in response to hemodynamic changes. Intravenous fluids were infused to maintain the central venous pressure at a minimum of 5 mmHg. Continuous infusion of inotropic drugs, such as dopamine, norepinephrine, and vasopressin, was administered to maintain mean arterial pressure at a minimum of 70 mmHg. Bolus injection of epinephrine was given when sudden hemodynamic instability was anticipated or had occurred. Indication for intraoperative transfusion of packed red blood cell was blood hemoglobin < 8.0 g/dL. To maintain normothermia, a warm blanket and a fluid warmer were used; the room temperature was thermostatically set at 24 °C.

The full blood test was immediately performed at the arrival into the ICU, and daily routine blood tests were performed during the hospital stay. Oxygenation, nutritional support, and early feeding and ambulation were encouraged while being closely monitored for early detection of postoperative complications such as bleeding, thrombosis, and biliary leakage or stricture.

### Statistical analysis

To divide the recipients according to NLR change during LDLT, we constructed six smooth plots of the change of OR for 1-year graft failure when the reference value was absolute NLR change of 0, median value of absolute NLR change, 80% of absolute NLR change, NLR percentage change of 0, median value of NLR percentage change, and 40% of NLR percentage change. NLR percent change defined as [postoperative NLR − preoperative NLR]/preoperative NLR. Differences between the groups for continuous data were compared using the t-test or the Mann–Whitney test when applicable and presented as mean ± standard deviation. Categorical data were presented numerically and compared by using *χ*^2^ or Fishers exact test. Kaplan–Meier estimates were used to construct survival curves for each group and compared with the log-rank test. We used a multivariable Cox regression analysis to adjust for variables between the two groups. Variables for the multivariable analysis were selected based on clinical relevance or *p* < 0.05. Retained variables include age, sex, MELD score, operative duration, alcoholics, hepatorenal syndrome, hypertension, preoperative ICU treatment, viral disease, cirrhotic disease, HCC, preoperative NLR > 4, donor sex, and donor age. The results were reported as HR with 95% CI. For sensitivity analysis, we estimated the potential impact of unmeasured confounders^29^. Considering the long study period, a sensitivity analysis was also performed for the former and latter cases. We stratified the recipients in a chronological order and divided them at the median number. The significance of the observed association between NLR change and 1-year graft failure was evaluated in recipients before and after November 2010.

Subgroup analysis was also conducted to reveal hidden interaction with relevant variables. To compare the impact of NLR decrease on graft failure with other variables, we calculated the AF for each variable based on the results of the Cox proportional hazards model^30^. This measure represents the proportional reduction of the outcome within a population that would occur if the incidence of the variable was reduced to zero. Multivariable logistic regression analysis was used to identify variables associated with NLR change during LDLT and was reported as odds ratio (OR) with 95% CI. The LASSO model, a regression analysis that fits a generalized linear model via penalized maximum likelihood and select variables, was used for more accurate evaluation^31^. All statistical analyses were performed with R 3.6.1 (Vienna, Austria; http://www.R-project.org/). All tests were 2-tailed, and *p* < 0.05 was considered statistically significant.

## Supplementary Information

Supplementary Informations.

## Abbreviations

NLR: Neutrophil–lymphocyte ratio
LDLT: Living donor liver transplantation
HCC: Hepatocellular carcinoma
ICU: Intensive care unit
