# Supplementary material for: Association between neutrophil–lymphocyte ratio change during living donor liver transplantation and graft survival
Source: Sci Rep. 2021 Feb 18;11:4199. doi: 10.1038/s41598-021-83814-9 (PMC7892541; doi:10.1038/s41598-021-83814-9)
Supplement: Supplementary file 1 — Supplementary Informations. [file 41598_2021_83814_MOESM1_ESM.docx]

**Association between neutrophil–lymphocyte ratio change during living donor liver transplantation and graft survival**

Jungchan Park, MD^1,†^, Seung-Hwa Lee, MD^2,†^, Mi Sook Gwak, MD^1^, Justin Sangwook Ko, MD^1^, Sangbin Han, MD^1^, Gyu-Seong Choi, MD^3^, Jae Won Joh, MD^3^, Jongman Kim, MD^3^, and Gaab Soo Kim, MD^1,*^

^1^Department of Anesthesiology and Pain Medicine, Samsung Medical Center, Sungkyunkwan University School of Medicine, Seoul, Korea

^2^Division of Cardiology, Department of Medicine, Heart Vascular Stroke Institute, Samsung Medical Center, Sungkyunkwan University School of Medicine, Seoul, Korea

^3^Department of Surgery, Samsung Medical Center, Sungkyunkwan University School of Medicine, Seoul, Korea

^†^J. Park and S.H. Lee contributed equally to this work.

Running title: Neutrophil–lymphocyte ratio change during living donor liver transplantation

^*^Corresponding author at: Gaab Soo Kim, MD, Department of Anesthesiology and Pain Medicine, Samsung Medical Center, Sungkyunkwan University School of Medicine, 81 Irwon-ro, Gangnam-gu, Seoul, Korea. 06351

Tel: +82-2-3410-0360; Fax: +82-2-3410-0361; E-mail address: gskim@skku.edu

Supplemental table 1. Causes of one-year graft failure according to the change of NLR

|  | Decrease (N=103) | Increase (N=1189) | P-value |
| --- | --- | --- | --- |
| Rejection | 1 (1.0) | 11 (9.3) | >0.99 |
| Cancer recurrence | 1 (1.0) | 24 (2.0) | 0.72 |
| Infection | 3 (2.9) | 11 (0.9) | 0.09 |
| Thrombosis | 2 (1.9) | 7 (0.6) | 0.16 |
| Bleeding | 5 (4.9) | 16 (1.3) | 0.02 |
| Biliary leakage | 1 (1.0) | 6 (0.5) | 0.44 |
| Biliary stricture | 0 | 2 (0.2) | >0.99 |
| Pulmonary | 3 (2.9) | 2 (0.2) | 0.004 |
| Cardiac | 3 (2.9) | 3 (0.3) | 0.01 |
| Cerebral | 0 | 5 (0.4) | >0.99 |
| Unknown | 5 (4.9) | 24 (2.0) | 0.08 |

NLR, neutrophil-to-lymphocyte ratio

Supplemental table 2. Sensitivity analysis of the effect of an unmeasured confounder on hazard ratio between intraoperative neutrophil-to-lymphocyte ratio decrease and graft failure

|  |  | OR*_ZY_*_\|_*_X_* | | | | | |
| --- | --- | --- | --- | --- | --- | --- | --- |
|  |  | 1.5 | 2 | 2.5 | 3 | 3.5 | 4 |
| OR_zx_ | 0.3 | 2.15 (1.25-3.69) | 2.38 (1.38-4.10) | 2.62 (1.51-4.52) | 2.70 (1.56-4.69) | 2.95 (1.71-5.08) | 3.01 (1.75-5.19) |
|  | 0.4 | 2.11 (1.23-3.62) | 2.27 (1.33-3.87) | 2.63 (1.53-4.50) | 2.24 (1.30-3.88) | 2.82 (1.64-4.84) | 2.86 (1.66-4.93) |
|  | 0.5 | 2.04 (1.19-3.48) | 2.18 (1.28-3.73) | 2.40 (1.41-4.11) | 2.43 (1.42-4.16) | 2.59 (1.52-4.42) | 2.45 (1.43-4.20) |
|  | 0.6 | 1.98 (1.17-3.36) | 2.08 (1.22-3.55) | 2.18 (1.29-3.68) | 2.14 (1.26-3.63) | 2.13 (1.24-3.65) | 2.29 (1.34-3.90) |
|  | 0.7 | 1.90 (1.12-3.22) | 2.15 (1.26-3.67) | 2.09 (1.22-3.58) | 2.54 (1.50-4.29) | 2.19 (1.28-3.75) | 2.55 (1.51-4.31) |

Prevalence of unmeasured confounder = 40%

Numbers represent HRs (including 95% CIs).

OR, odds ratio; HR, hazard ratio; X: dichotomous exposure measure, y dichotomous outcome measure, z: potential dichotomous confounder.

OR_ZX_ indicates the association (OR) between the unmeasured confounder and intraoperative neutrophil-to-lymphocyte ratio decrease.

OR_ZY|X_ indicates the association (OR) between the unmeasured confounder and graft failure conditional on exposure status.

Supplemental table 3. Sensitivity analysis on hazard ratio of NLR decrease for one-year graft failure

|  | OR (95% CI) | P-value |
| --- | --- | --- |
| Recipients before November 2010 (n=646) | 2.32 (1.33-4.04) | 0.003 |
| Recipients after November 2010 (n=646) | 3.68 (1.67-8.12) | 0.001 |

NLR, neutrophil-to-lymphocyte ratio; HR, hazard ratio; CI, confidence interval
